# Transcriptome profiling reveals candidate flavonol-related genes of *Tetrastigma hemsleyanum* under cold stress

**DOI:** 10.1186/s12864-019-6045-y

**Published:** 2019-08-31

**Authors:** Xin Peng, Hao Wu, Hongjiang Chen, Yujiong Zhang, Dan Qiu, Zhongyi Zhang

**Affiliations:** 1 Institute of Biopharmaceutical Technology, Zhejiang Pharmaceutical College, Ningbo, 315100 Zhejiang, People’s Republic of China; 2 Fujian Agriculture and Forestry University, Fuzhou, 350000 Fujian People’s Republic of China; 3 Ningbo Engineering College, Ningbo, 315100 China

**Keywords:** *Tetrastigma hemsleyanum*, Transcriptome, Cold stress, Flavonol

## Abstract

**Background:**

*Tetrastigma hemsleyanum* Diels et Gilg is a valuable medicinal herb, whose main bioactive constituents are flavonoids. Chilling sensitivity is the dominant environmental factor limiting growth and development of the plants. But the mechanisms of cold sensitivity in this plant are still unclear. Also, not enough information on genes involved in flavonoid biosynthesis in *T. hemsleyanum* is available to understand the mechanisms of its physiological and pharmaceutical effects.

**Results:**

The electrolyte leakage, POD activity, soluble protein, and MDA content showed a linear sustained increase under cold stress. The critical period of cold damage in *T. hemsleyanum* was from 12 h to 48 h. Expression profiles revealed 18,104 differentially expressed genes (DEGs) among these critical time points. Most of the cold regulated DEGs were early-response genes. A total of 114 unigenes were assigned to the flavonoid biosynthetic pathway. Fourteen genes most likely to encode flavonoid biosynthetic enzymes were identified. Flavonols of *T. hemsleyanum* might play a crucial role in combating cold stress. Genes encoding P*AL, 4CL*, *CHS, ANR, FLS*, and *LAR* were significantly up-regulated by cold stress, which could result in a significant increase in crucial flavonols (catechin, epicatechin, rutin, and quercetin) in *T. hemsleyanum.*

**Conclusions:**

Overall, our results show that the expression of genes related to flavonol biosynthesis as well as flavonol content increased in *T. hemsleyanum* under cold stress. These findings provide valuable information regarding the transcriptome changes in response to cold stress and give a clue for identifying candidate genes as promising targets that could be used for improving cold tolerance via molecular breeding. The study also provides candidate genes involved in flavonoid biosynthesis and may be useful for clarifying the biosynthetic pathway of flavonoids in *T. hemsleyanum*.

**Electronic supplementary material:**

The online version of this article (10.1186/s12864-019-6045-y) contains supplementary material, which is available to authorized users.

## Background

*Tetrastigma hemsleyanum* Diels et Gilg is a valuable and endangered perennial medicinal herb, which is distributed in tropical to subtropical regions, mainly in the few provinces of southwest China. Extracts of total flavonoids from *T. hemsleyanum* have obvious antibacterial, antiviral, antitumor, and antipyretic actions [1]. Due to over exploitation, wild resources are on the verge of extinction in recent years. *T. hemsleyanum* is a cold-sensitive species, and chilling sensitivity is the major environmental factor limiting growth, development and geographical distribution of the plant. The artificial domestication of *T. hemsleyanum* suffers from the low winter survival rate.

Cold stress is a major environmental factor that influences plant growth, development, and productivity. In order lead to adapt to cold stress during acclimation, plants have evolved various adaptive mechanisms that trigger gene-expression changes and subsequently to physiological and biochemical modifications that enhance their cold tolerance. The stress responses include: increase in antioxidant levels, induction of influx of cellular calcium ions, alteration in membrane lipid composition, adjustment of hormone levels, changes in electrolyte leakage and soluble protein content, etc. [2].

Genome-wide transcriptome analysis provides a potentially valuable strategy for identifying candidate genes for molecular breeding and for revealing the molecular mechanisms underlying physiological processes. Recent developments in deep-sequencing technologies and relevant analytical approaches can provide the large-scale transcriptome information in the absence of a reference genome, and thus it is especially useful in non-model species, whose genomic sequences are often unavailable [3]. Over the last few years, RNA-Seq has been increasingly applied to rapidly analyze mRNA profiles in a range of plant species under exposure to diverse and adverse environmental conditions, including drought [4], high salinity [5], heavy metal stress [6],pathogen attack, and cold stress [7]. It allows us to use a “reverse genetics” strategy to identify candidate genes, to provide available gene resources for developing transgenic plants. Early plant responses to chilling stress involve numerous genes encoding transcription factors and proteins involved in phytohormone signal transduction, ROS metabolism, secondary metabolism, etc. [8].

Flavonoids are a large class of plant secondary metabolites that exert diverse biological and pharmacological effects, including the protection of plants against abiotic stresses. The major bioactive constituents in *T. hemsleyanum* are flavonoid compounds. To date, more than 20 flavonoids have been isolated from it. Flavonoids compounds can be divided into six categories: flavonoids, flavonols, isoflavones, flavanones, flavanols, and anthocyanins, among which flavonols are the main compounds in the *T. hemsleyanum*. Our previous studies indicated that the main flavonols in *T. hemsleyanum* were quercetin, procyanidine B1, procyanidine B2, catechin, epicatechin, rutin, kaempferol etc. However, gene regulation and signaling pathways related to cold response in *T. hemsleyanum* remain unknown. Not enough information on genes involved in flavonoid biosynthesis in *T. hemsleyanum* is available to understand the mechanisms of its physiological and pharmaceutical effects, and there still has not been any report about flavonoid metabolomic profiling of *T. hemsleyanum* response to environmental stresses.

Our aim was therefore to elucidate the physiological and transcriptomic adaptive mechanisms to cold stress in *T. hemsleyanum* by using comparative transcriptome analyses to evaluate the global gene dynamic gene expression patterns. Based on this, genome-wide characterization of flavonoid biosynthetic genes in *T. hemsleyanum* and their relevance to cold stress tolerance would be revealed. The results will facilitate the identification of cold-resistant candidate genes in *T. hemsleyanum*, which could be used for genetic improvement. The results will also shed light on the molecular mechanisms related to cold tolerance and pharmaceutical effects in this plant.

## Results

### Physiological changes of *T. hemsleyanum* under cold stress

When exposed to − 8 °C or − 4 °C, the seedlings of *T. hemsleyanum* exhibited severe damage within 4 h and died within 8 h. When exposed to 4 °C, there was no significant leap in electrolyte leakage during stress treatment. Therefore, we selected the 0 °C exposure for cold stress treatment. To estimate the physiological damage and to optimize the sampling time of cold treatment, four physiological parameters, including MDA, soluble protein content, POD activity, and electrolyte leakage were measured under 25 °C or 0 °C exposure for seven time points (0, 4, 8, 12, 24, 48, and 72 h) in leaves, with three biological replicates.

**Fig. 1:**
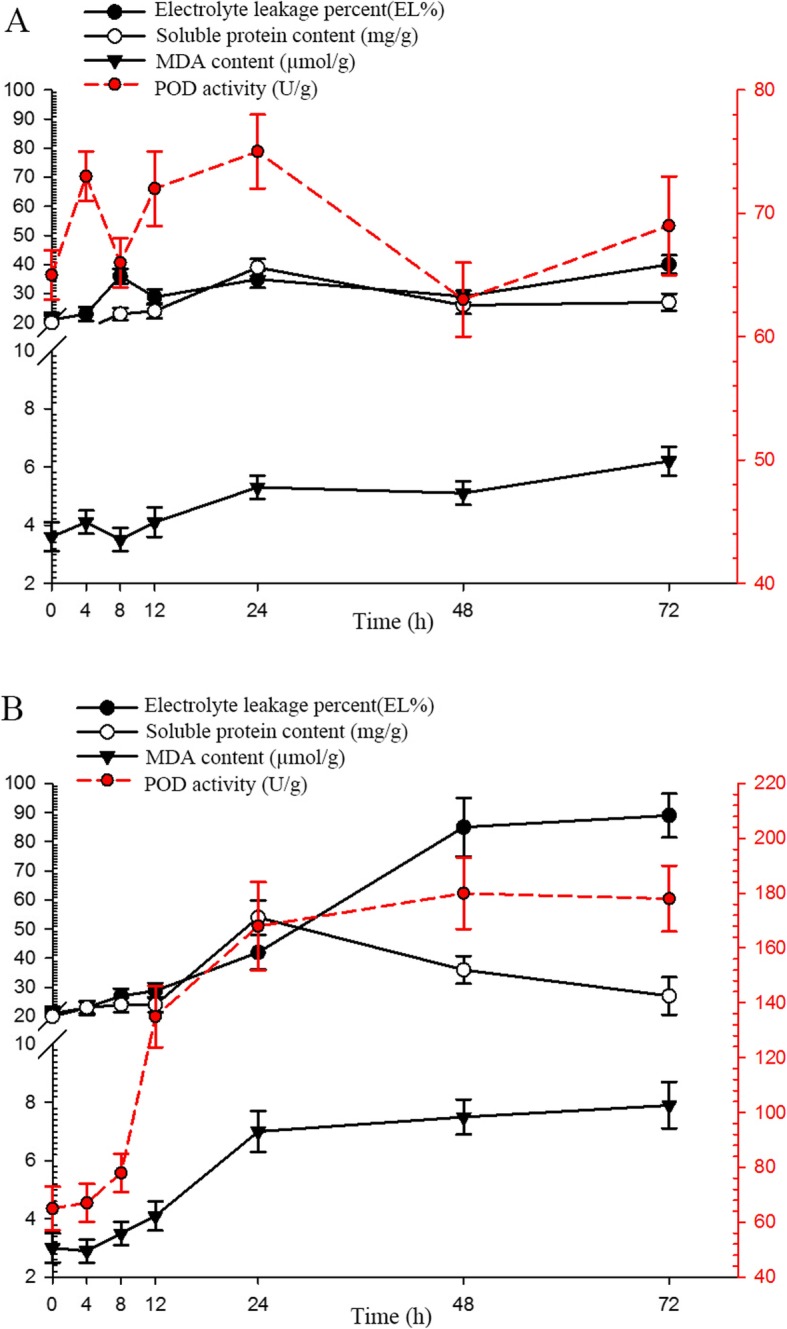
Physiological changes of *T. hemsleyanum* leaves under 25 °C (**a**) or 0 °C (**b**) exposure across seven time points (0, 4, 8, 12, 24, 48, 72 h)

As seen from Fig. 1, four physiological parameters were almost unchanged across different time points at 25 °C. The results showed that as the treatment time at 0 °C was prolonged, electrolyte leakage increased dramatically from 21.3 ± 2.4%(0 h) to 89.7 ± 7.5%(72 h) (*P* < 0.01). There was a sudden and sharp increase at the time point from 12 h to 48 h, and remained stable after 48 h. Similarly, MDA content also exhibited a linear increase from 3.1 ± 0.4 μmol/g (0 h) to 7.9 ± 0.8%(72 h) (*P* < 0.05). There was a sudden and sharp increase at the time point from 12 h to 24 h, and a steady increase after 24 h. Soluble protein content also showed steady increase during 0 h to 24 h, it showed a high peak at 24 h and then declined from 54 ± 5.3 mg/g(24 h) to 27 ± 3.3 mg/g(72 h) (*P* < 0.05). However, there was a sudden and sharp increase of POD activity at the time point from 8 h to 24 h(*P* < 0.01), and remained stable after 24 h.

In summary, the contents/activities of the four physiological parameters remained essentially stable during 0 h and 8 h, significantly changed from 12 h to 48 h, and remained stable after 48 h. To further explore the mechanism of cold stress, RNA-seq was employed to investigate the changes in genome-wide gene expression in *T. hemsleyanum* leaves under 0 °C exposure for four time points (0, 12, 24, and 48 h), with three biological replicates.

### De novo assembly and annotation

**Fig. 2:**
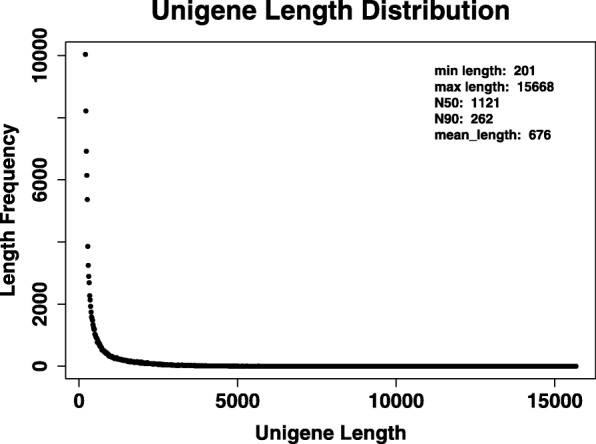
Length distribution of transcripts in *T. hemsleyanum* transcriptome

Using a total of 54,433,014; 54,981,362; 53,390,246; 53,238,442 Illumina raw reads from four independent biological samples (0, 12, 24, and 48 h), we got a total of 45,346,334; 46,460,402 44,804,246; 44,801,200 clean reads, with an average clean reads rate of 83.31, 84.5, 83.92, and 84.15%. These high-quality reads were then de novo assembled using the Trinity program [9], resulting in 151,924 ‘Trinity’ genes and 106,275 unigenes, which ranged from 201 to 15,668 bp in length. The average length of unigenes was 676 bp. The N50 and N90 length was 1121 bp and 262 bp, respectively. This indicates a high quality assembly (Additional file 1). Of these unigenes, 48,464 (55.4%) were longer than 500 bp, 19,627 (18.5%) were longer than 1000 bp, 6946 (6.53%) were longer than 2000 bp, etc. The length distribution of transcripts is shown in Fig. 2.

**Fig. 3:**
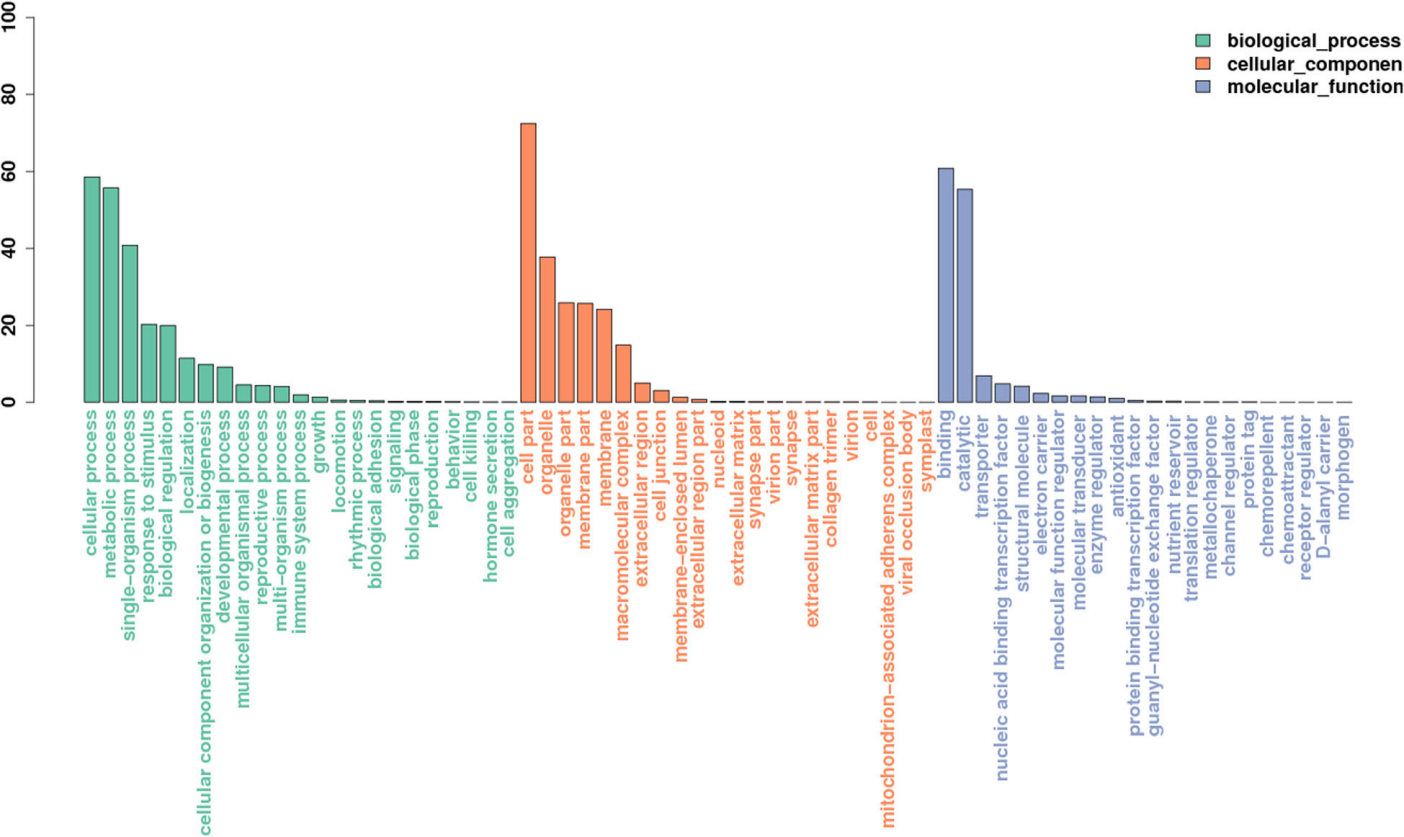
Gene ontology classification of the *T. hemsleyanum* transcriptome

To validate and annotate the of assembled unigenes, they were blast searched against seven public databases, including NT, NR, Uniprot database, RNAMMER, eggnog, GO, and KEGG using E-values <1e-5. To characterize the functional classifications of annotated unigenes, GO and COG analyses were performed to access the functional categories. Based on sequence homology, a total of 35,665 (40.7%) unigenes were annotated in GO, and classified into 67 functional groups, including 23 groups in biological process, 23 in cellular components, and 21 in molecular function (Fig. 3). ‘Cell part’, ‘organelle’, and ‘organelle part’ were the terms that dominated in the cellular component category. ‘Cellular process’, ‘metabolic process’, ‘single-organism’, and ‘response to stimulus’ were the most represented GO terms in the biological process category. Most of the unigenes in the ‘molecular function’ category were sub-categorized into ‘binding’ and ‘catalytic’, followed by ‘transporter’. In the ‘cellular component’ category, the GO terms ‘cell part’, ‘organelle’, and ‘membrane part’ predominated.

**Fig. 4:**
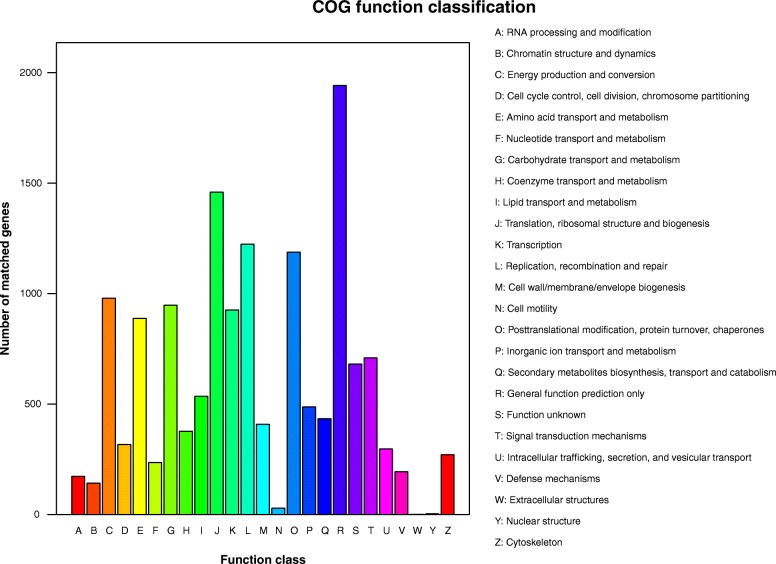
COG functional classification of the *T. hemsleyanum* transcriptome

All unigenes were matched with the Cluster of Orthologus Groups (COG database) for functional prediction and classification. In total, 82,192 (40.99%) unigenes were assigned to 25 COG functional classifications. These unigenes were mainly involved in ‘general function prediction only’, ‘translation, ribosomal structure and biogenesis’, and ‘replication, recombination and repair’(Fig. 4).

### Gene expression changes triggered by cold stress

**Fig. 5:**
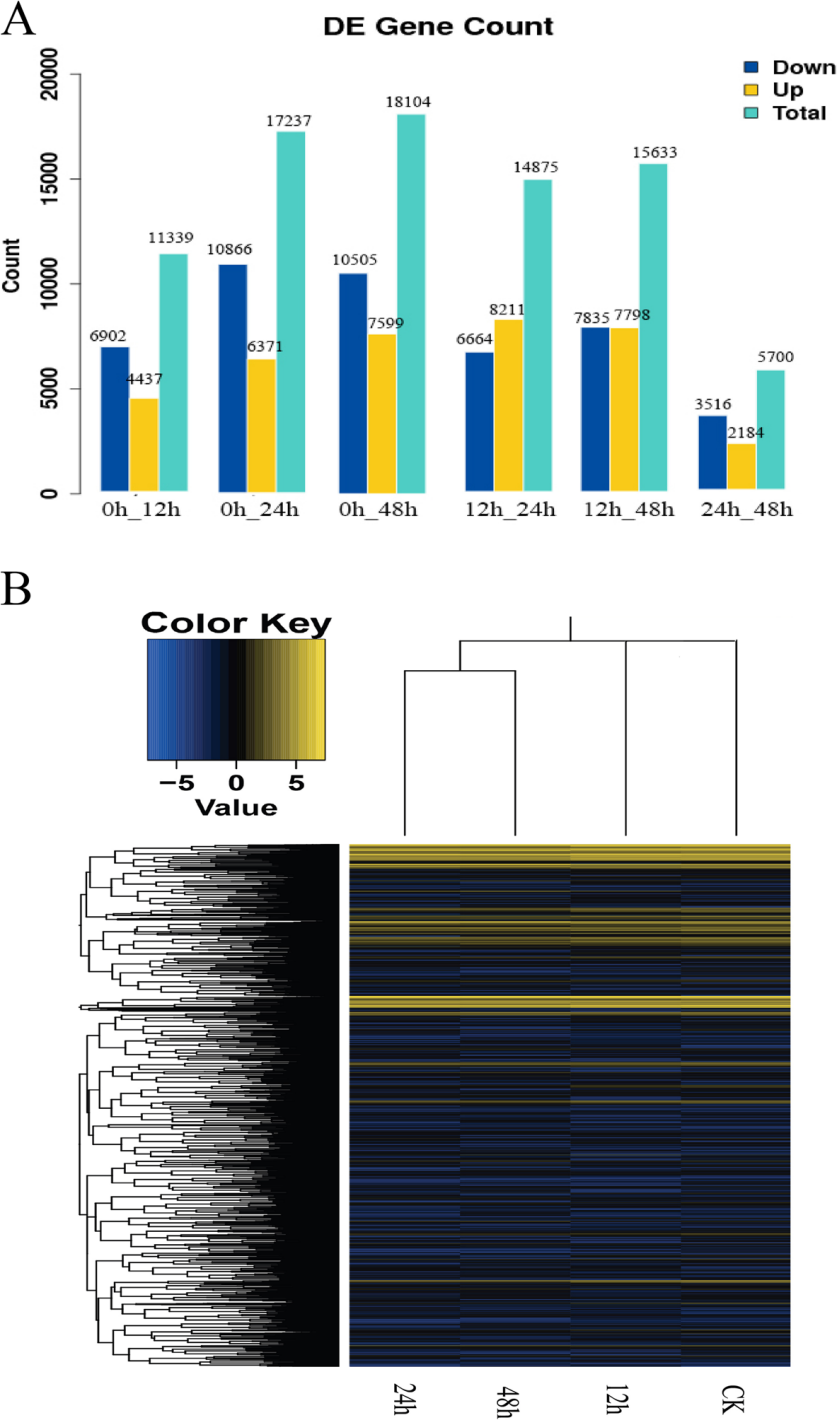
Transcriptional profiles of *T. hemsleyanum* after 0 to 48 h of cold exposure. **a** Distribution of genes differentially expressed under 0 °C exposure for 12 h, 24 h, and 48 h of cold exposure, **b** Hierarchical clustering analysis of the commonly expressed DEGs from the RNA-Seq in each treatment (yellow, induced genes; blue, repressed genes)

Differential gene expression was analyzed using all control and treatment combinations (Fig. 5a). Over the 12 h, 24 h, 48 h of cold exposure, a total of 11,339, 17,237, and 18,104 DEGs were identified and analyzed, respectively. Of those genes, about 6902 (60.8%), 10,866 (63.0%), 10,505 (58.0%) were down-regulated, while the rest of genes were up-regulated respectively. Most of the cold regulated DEGs were early-response genes. There were 14,875 and 15,633 unigenes differentially expressed in the 12 h–24 h and 12 h–48 h comparisons, respectively, while only 5722 induced/repressed unigenes were specific between 24 h and 48 h of cold exposure. Of those genes, about 6664 (44.8%), 7835 (50.1%), 356 (61.7%) were down-regulated, while the rest of genes were up-regulated respectively.

A hierarchical cluster analysis was performed to characterize the expression patterns of the commonly expressed DEGs after cold exposure (Fig. 5b). Most of DEGs followed a similar expression pattern after 24 h and 48 h of cold exposure, which clustered together. In contrast, expression profiles of 0 h (control) and 12 h of cold exposure clustered separately. It indicated that there were obvious differences in the patterns of global gene expression at early and later phases of cold stress.

### Responses of flavonoid metabolism-related genes

GO and KEGG analyses were performed to access the distributions of functional categories. ‘response to stimulus’, ‘response to stress’, and ‘membrane’were the most abundant terms in functional category during the whole cold stress. ‘metabolic pathways’, ‘biosynthesis of secondary metabolites’, and ‘phenylpropanoid biosynthesis’ were the top 3 enriched pathways during the whole cold stress stage. Based on the above overview of GO and KEGG analyses, we speculated that phenylpropanoid metabolism would play an important role in early-time responses of *T. hemsleyanum* under cold exposure. Flavone and flavonol are main component of *T. hemsleyanum* and also metabolites of phenylpropanoid metabolism. So we mainly focused on groups of differentially expressed genes involved in flavonoid biosynthesis pathways.

**Fig. 6:**
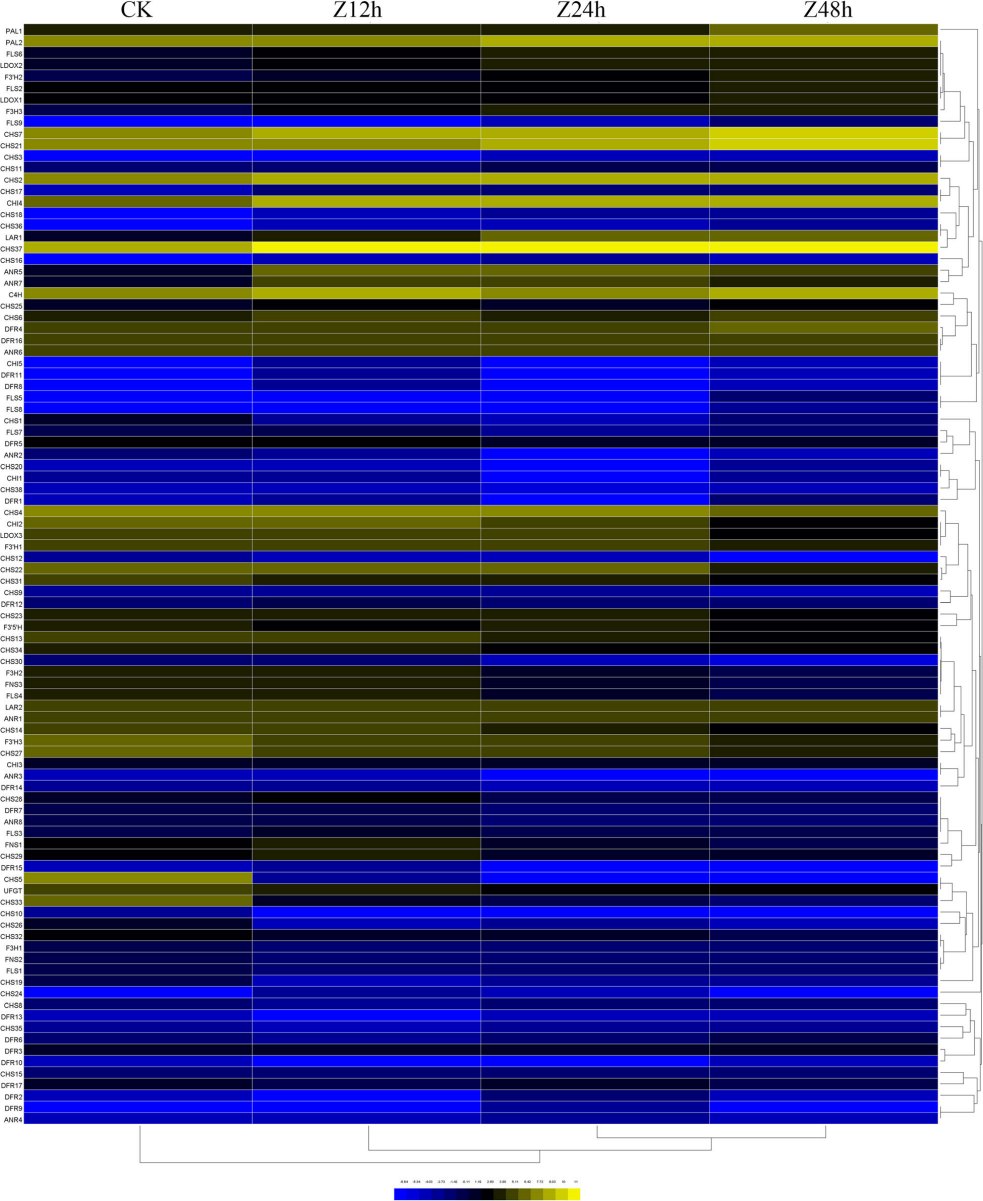
Heatmap of the overall changes of flavonoid metabolism-related genes in *T. hemsleyanum* after 0 to 48 h of cold exposure

Using the *T. hemsleyanum* transcriptome with its annotated information, we investigated all the genes involved in flavonoid biosynthesis at the level of whole genome expression level. Transcriptome analysis detected 114 unigenes encoding multiple well-known enzymes in the flavonoid biosynthesis and metabolism pathway. From Fig. 6, we could see that *T. hemsleyanum* included almost all of the necessary enzymes involved in the general pathway, such as phenylalanine ammonia-lyase (PAL), flavanone 3-hydroxylase(F3’H), cinnamate-4-hydroxylase (C4H), 4-coumaroyl:coenzyme A ligase (4CL), chalcone synthase (CHS), flavanone-3-hydroxylase (F3H), flavonol synthase (FLS) etc. Most of flavonoid metabolism-related genes followed a similar expression pattern after 24 h and 48 h of cold exposure, thus they clustered together. In contrast, expression profile of 0 h (control) and 12 h of cold exposure clustered separately. The clustering method was in accordance with that of the global DEGs after cold exposure.

**Fig. 7:**
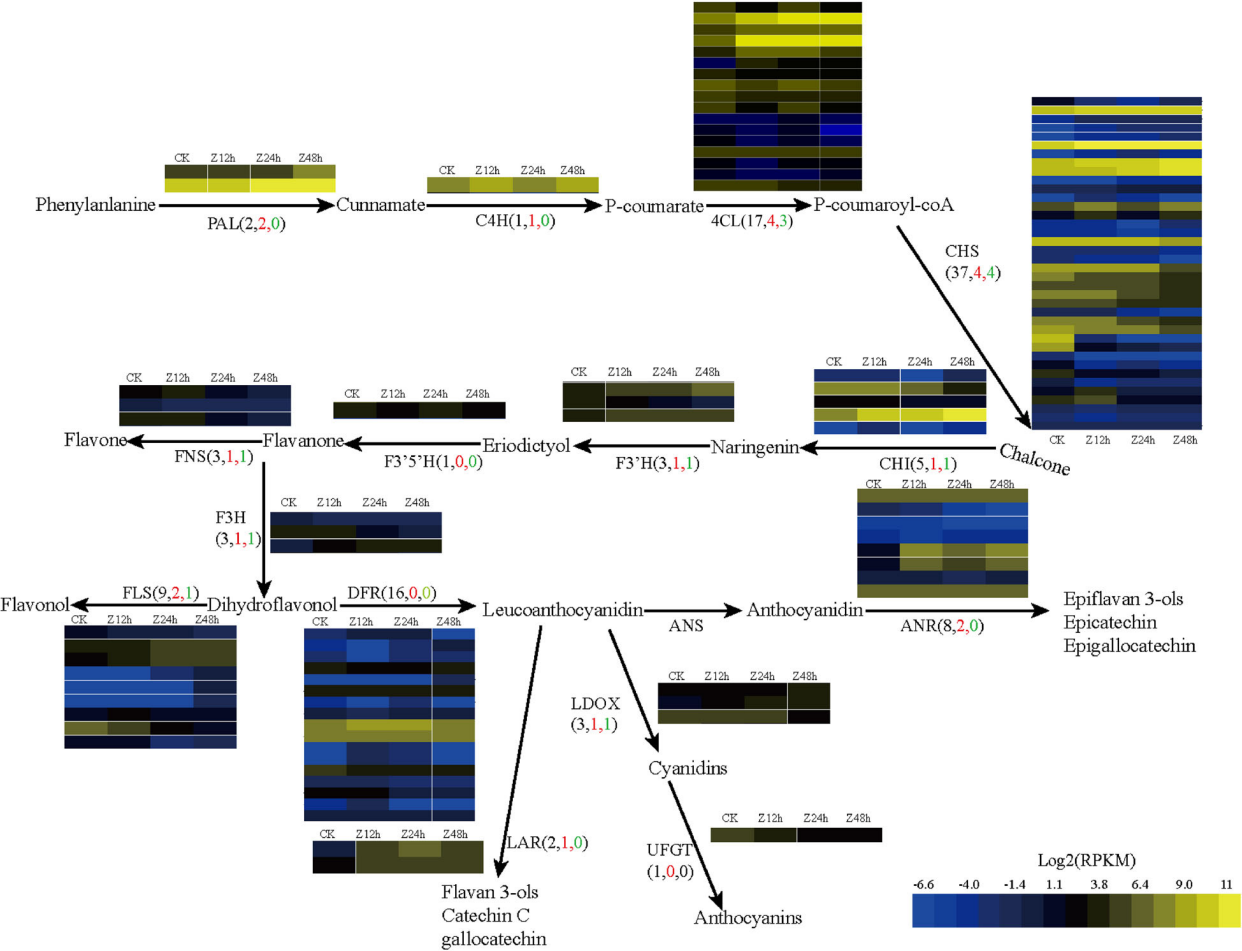
The schematic diagram of the flavonoid biosynthetic pathways. The heatmap presents the expression levels of flavonoid biosynthetic genes based on *T. hemsleyanum* RNA sequencing data. The first number in brackets following each gene name refers to the number of transcriptome unigenes annotated as that gene, while the red and green numbers on the arrows indicated the number of significantly up-regulated and down-regulated unigenes, respectively. A color bar is presented at the bottom right. Data represent the log2 values (RPKM) of the *T. hemsleyanum* during the CK, Z12h, Z24h, and Z48h stages (from left to right)

The schematic diagram of the flavonoid biosynthetic pathways and the expression levels of related genes in *T. hemsleyanum* are displayed in Fig.7. PAL catalyzes the initial step of the flavonoid biosynthetic pathway. Among the mapped enzymes, we identified two PAL homologs in annotated transcriptome unigene dataset, and both of them were highly expressed with RPKM values> 100 and were significantly up-regulated by 3–4 fold under cold stress. One highly expressed C4H gene was predicted in our dataset, which was significantly up-regulated by 2.5 folds under cold stress. These genes encode the enzyme that catalyzes the second step of the flavonoid biosynthetic pathway. Thirty-seven CHS unigenes were identified in the *T. hemsleyanum* transcriptome. CHS is also the key enzyme in the flavonoids synthesis pathway, catalyzing the process from malonyl-CoA to naringenin chalcone. The expression of the 22 CHS genes was almost undetectable in the four analyzed libraries. The remaining 15 CHS unigenes were highly expressed, among which c101781_g1 was the most highly expressed CHS gene, with an increased RPKM value from 923 to 7889 under cold stress. Five CHI genes were identified in our dataset, and the fact of c82212_g1 was the most highly expressed suggested it may be crucial for naringenin biosynthesis, with an increased RPKM value from 128 to 988 under cold stress. In contrast, only three F3′H, one F3′5’H, three FNS, and three F3H genes were predicted in our dataset. ANR and LAR catalyze the transfer of leucoanthocyanidin to the flavonols group, such as epicatechin, epigallocatechin, catechin C, gallocatechin, etc. C78251_g1 was the most highly expressed LAR gene, with a 39 fold significant up-regulation under cold stress. Eight ANR genes were identified, with c84306_g1 and c78251_g1 being the most abundantly and the most remarkably differentially expressed. Most of these genes were highly expressed at Z12h and Z24h, but exhibited down-regulated expression at Z48h. These results imply the early and late stages of cold stress might be important for flavonoid biosynthesis.

A color bar is presented at the bottom. Data represent the log2 values (RPKM) of the *T. hemsleyanum* during the CK, Z12h, Z24h, and Z48h stages (from left to right). The trees on the right were generated using the Cluster 3.0 program.

### Validation of the differentially expressed genes (DEGs) via qRT-PCR

**Fig. 8:**
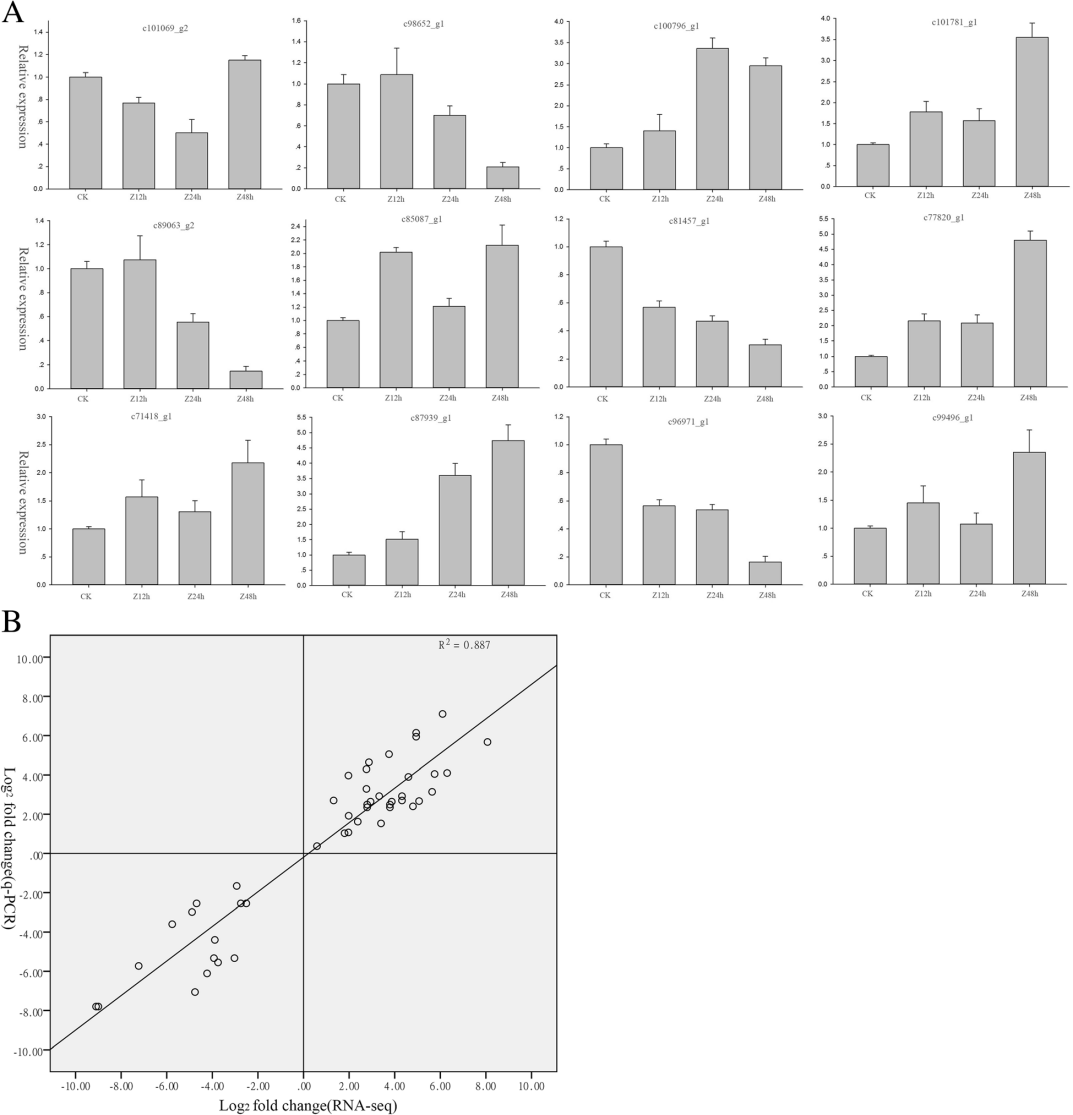
The expression of favonol-related genes of *T. hemsleyanum* after 0 to 48 h of cold exposure. **a** The genes expression as determined by qRT-PCR. **b** Comparison between the log2 of gene expression ratios obtained from RNA-seq data and qRT-PCR. The qPCR log2value of the expression ratio (cold-acclimated/CK) (y-axis) was plotted against the value from the RNA-seq (x-axis). All qPCR data were collected from three biological replicates and three technical replicates for each sample

To verify the reliability and accuracy of this Illumina RNA-seq data, quantitative real-time reverse transcription-PCR (qRT-PCR) was used to assess the relative expression levels for 12 key genes in the conserved flavonoid biosynthesis pathway in every treatment group. GAPDH was selected as the internal control reference gene. The correlation between qRT-PCR and RNA-seq was measured by scatter plotting log2-fold changes, which showed a positive correlation coefficient in both techniques (Pearson coefficient *R*^2^ = 0.88) (Fig. 8). This independent evaluation revealed the reliability of the RNA-seq data. The results showed that the expression of upstream genes, such as P*AL, 4CL*, and *CHS* were usually higher than the other downstream genes in flavonoid biosynthesis (shown in Fig. 7). These above key upstream genes and some of downstream genes for flavonols synthesis, such as *ANR, FLS*, and *LAR*, were significantly up-regulated under cold stress. Moreover, according to the deep sequencing and qRT-PCR data, it was suggested that flavonols of *T. hemsleyanum* might play a crucial role in combating cold stress.

### Analysis of various flavonols components

**Fig. 9:**
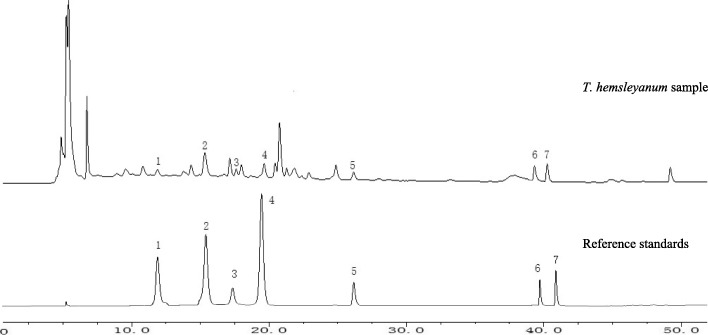
The HPLC chromatograms of reference standards and *T. hemsleyanum* sample. 1 procyanidins B_1_; 2. catechin; 3. procyanidinsB_2_; 4. epicatechin; 5. rutin; 6. quercetin; 7. kaempferol

The content of seven flavonols (procyanidins B1, catechin, procyanidinsB2, epicatechin, rutin, quercetin, and kaempferol) were determined by HPLC analysis. The HPLC chromatograms of reference standards and *T. hemsleyanum* sample are shown in Fig.9. Seen from Table 1, epicatechin was the most abundant flavonol (average content 12.48 ± 1.80 mg/g), followed by rutin (average content 10.32 ± 1.51 mg/g) and catechin (average content 5.43 ± 0.8%). This demonstrated that the total contents of most flavonols in stress groups were much higher than those in control group. The content of individual flavonols increased with the increase of cold stress duration. The levels of catechin, epicatechin, rutin, and quercetin in stress groups were higher than those detected in CK, ranging from 2.93 ± 0.3 (Z12h) to 5.13 ± 0.9 (Z48h) times, 3.08 ± 0.6 (Z12h) to 5.28 ± 0.8 (Z24h) times, and 2.71 ± 0.2 (Z12h) to 3.68 ± 0.5 (Z48h) times, and 2.61 ± 0.3 (Z12h) to 4.92 ± 0.5 (Z48h) times, respectively. The contents of other compounds of flavonols were also evaluated, but the differences were not significant.

This tendency was in accordance with the change in flavonol synthesis-related gene expression induced by cold stress (shown in Table 1). Therefore, these results suggested that the overexpression of key genes in flavonol biosynthesis (such as *ANR*, *FLS*, and *LAR)* induced by cold stress would result in a significant increase in crucial flavonol ingredients (such as catechin, epicatechin, rutin,and quercetin) in *T. hemsleyanum.*

**Table 1:** Each flavonol content in *T. hemsleyanum* under cold stress for 0 h (CK), 12 h (Z12h), 24 h(Z24h), and 48 h(Z48h)

Sample	Each flavonol content (mg·g^−1^ DW. ± SE)						
	Procyanidins B1	Catechin	Procyanidins B2	Epicatechin	Rutin	Quercetin	Kaempferol
CK	2.63 ± 0.15^a^	1.64 ± 0.085^a^	0.06 ± 0.005^a^	3.52 ± 0.19^a^	3.80 ± 0.19^a^	0.69 ± 0.045^a^	0.48 ± 0.031^a^
Z12h	2.91 ± 0.19^a^	4.73 ± 0.30^b^	0.11 ± 0.006^a^	10.80 ± 0.78^b^	10.3 ± 0.67^b^	1.80 ± 0.11^b^	0.60 ± 0.047^a^
Z24h	4.52 ± 0.28^b^	7.21 ± 0.39^c^	0.12 ± 0.008^a^	18.52 ± 1.32^c^	13.2 ± 0.98^b^	3.11 ± 0.21^c^	0.71 ± 0.052^a^
Z48h	4.65 ± 0.32^b^	8.22 ± 0.51^c^	0.09 ± 0.078^a^	17.13 ± 1.36^c^	14.21 ± 1.08^b^	3.41 ± 0.32^c^	0.52 ± 0.045^a^

Different English letters in the same column indicate a significant difference through pairwise comparison by LSD multiple comparison test (*P* < 0.05)

## Discussion

### The physiological responses of *T. hemsleyanum* to cold stress

Freezing injury is one of the most universal stresses influencing plant metabolism. The membrane permeability is often used as an important physiological index of plant resistance. The higher the electrolyte leakage rate is, the more serious the damage by cold stress is. The extent of electrolyte leakage is inversely correlated with the stress tolerance of plants. Under cold stress conditions, various osmotic substances such as proline and soluble proteins that play important roles in osmotic adjustment are quickly accumulated [10]. Besides, Malandialdehyde (MDA) is the final product of lipid peroxidation, its content can reflect the severity of membrane lipid peroxidation and the stress tolerance of plants. Antioxidative enzymes such as peroxidase (POD) and superoxide dismutase (SOD) are induced quickly and required to deal with oxidative damage under cold stress [11]. Thus, the content of these molecules and the activity of these enzymes are widely used as parameters to evaluate the characteristics of plants when they are suffering from stress.

The physiological responses of *T. hemsleyanum* to cold stress have not been extensively explored. In this study, four traits including electrolyte leakage, POD activity, the content of MDA and soluble protein were investigated in *T. hemsleyanum* leaves at seven time points under four low-temperature treatments. Similar to previous studies in other species, these investigated traits revealed a dramatic induction in response to cold treatment, and the effect was aggravated along with the prolonging of stress time. The electrolyte leakage, POD activity, and MDA content showed a linear sustained increase as treatment time went up from 0 h to 72 h, by 321.4 ± 15.3%, 173.8 ± 10.2%, and 163.3 ± 12.1%, respectively. During this period, 12 h, 24 h, and 48 h were critical time points of the dramatic change. The three traits exhibited a consistent response, indicating they played highly coordinate functions in *T. hemsleyanum*. However, it is worth noting that the tendency of soluble protein content was slightly different from the above three traits. The soluble protein content increased by 171.0 ± 9.2% from 0 h to 24 h, then there was a significant decline after that time point. Similarly, the content of soluble protein and soluble sugar of *L. lancifolium* leaves increased between 0 h to 24 h, then rapidly fell during subsequent cold stress [12]. It might be because that some stress proteins in plant signal transduction were induced in the early stress stage in responses to environmental stresses. Along with the prolonging of stress time, the cellular damage and apoptosis might accelerate protein degradation after 24 h. So 12 h and 24 h were critical time points of the dramatic change of soluble protein content. To obtain insights into the molecular mechanisms of the cold stress, a whole-transcriptome analysis induced by cold stress at these critical time points in *T. hemsleyanum* was then performed.

With the application of next-generation sequencing technology, studies of plant functional genomics have been rapidly performed in recent years. In this way, the DEGs obtained at different physiological states could provide useful information to clarify the molecular mechanisms underlying physiological changes. In this study, the hierarchical cluster analysis of the DEGs indicated that most of differential gene expression changes occurred in early stress stage, then remained relatively stable in middle and late phases of cold stress. These results also demonstrated that the global gene expression patterns coincided with the changes in physiological traits, indicating that these early emergency response genes play important roles in cold-acclimated of *T.hemsleyanum.*

### Identification of genes critical for flavonoid biosynthesis in *T. hemsleyanum* to cold stress

Flavonoids are important secondary metabolites that contain various important bioactive substances. Several subclasses of flavonoids, including flavonols and anthocyanidins, have been considered to be important antioxidants and have been associated with various biological effects including prevention of age-related diseases [13] and cancers of humans and animals [14]. Flavonoids are also produced as a protective response element in stress resistance-responsive system. Flavonoid biosynthesis is induced under a wide range of abiotic stresses. They could protect the plant from the heavy metal stress [15], nutrient starvation [16], and other environmental stresses. For example, in *Reaumuria trigyna*, the expressions of genes related to the flavonol biosynthesis were induced by ultraviolet-B and salt stresses [9], and many studies have shown that flavonol synthesis can protect plant from high-temperature damage [17]. To date, the effects of abiotic stress on the biosynthesis of flavonoids have been investigated mainly in crop and model plants. Comparatively few relative studies have been reported in medicinal plant.

Total flavonoids from *T. hemsleyanum* have been shown to be closely associated with its health promoting effects, including antioxidant, anticancer, and anti-inflammatory activity [18]. Investigation on the influence mechanism of cold stress on flavonoid metabolism pathways is particularly important. In the present study, we identified the major genes associated with flavonoid biosynthesis and accumulation in *T. hemsleyanum*. A total of 114 unigenes in our transcriptome dataset were assigned to the flavonoid biosynthetic pathway. With the exception of F3′5′H and UFGT, most of these enzymes were encoded by more than one annotated gene. Such sequences may represent different fragments of a single transcript, different members of a gene family, or both. Similarly, in the transcriptome data of NaCl-treated *Reaumuria trigyna*, 118 unigenes were identified to be flavone synthetase genes. Among these 118 unigenes, the expression levels of genes related to the flavonol biosynthesis pathway (*C4H*, *CHS*, and *FLS*) were higher than other flavone synthetase genes [9]. However, in the previously annotated *C. chekiangoleosa* transcriptome dataset [19], Forty-three unigenes were annotated as flavone synthetase genes, among which, F3H, DFR, and UFGT were much more actively involved in flavonoid biosynthesis than these genes in our study. The reason for the difference may be because anthocyanin and flavonol are two different subclasses of flavonoids, and they are generated in different branches of flavonoid synthesis pathway. The major component of cold-treated *T. hemsleyanum* and NaCl-treated *Reaumuria trigyna* were both flavonol, while the major component of *Camellia chekiangoleosa* was anthocyanin. Thus, this pathway appears to be conserved among different species, but there is still some diversity.

UV-B, C irradiation significantly promoted anthocyanin biosynthesis and the transcripts of anthocyanin downstream pathway genes (*DFR*, *ANR*, and *UFGT*) as well as *DFR* and *UFGT* enzymatic activities in blueberries [20]. In our study, flavonols content was positively correlated with the transcripts of downstream genes (*LAR*, *ANR*, and *LDOX*) as well as initiative upstream genes (*PAL* and *C4H*) in flavonoid biosynthetic pathway responding to cold stress and coincided with flavonols biosynthesis. The content of five flavonols in *T. hemsleyanum* including procyanidins B1, catechin,epicatechin, rutin, and quercetin remarkably increased in response to cold stress. Similar studies involving *Reaumuria trigyna* have also shown that flavonol biosynthesis was induced by ultraviolet-B and salt stresses. Of 118 annotated flavone synthetase unigenes, 47 were annotated as members of families related to the flavonol biosynthetic pathway. The expressions of genes related to the flavonol biosynthesis pathway (*C4H, CHS, F3H3, FLS, F3’5’H, F3’H*, and *OMT*) increased under NaCl and UV-B treatments. The content of several flavonols including rutin, hyperoside, isorhamnetin-3-O-neohespeidoside, and myricetin increased in response to NaCl and UV-B stresses [9]. In grape berries, UV-B increased the expression of the key flavonol biosynthesis gene *FLS, F3′H, F3′5′H*, and *OMT*, resulting in increased flavonol and anthocyanin content in the skin [21]. The higher transcript levels of *FLS* and a higher amount of 3 main flavonols (kaempferol, myricetin and quercetin) were observed in tartary buckwheat under different environmental stress [22].

*FLS*, an important enzyme in flavonoid biosynthesis pathway, catalyzes the oxidizing reaction of dihydroflavonol to form flavonols (e.g., quercetin, kaempferol, and Myricetin). Quercetin and kaempferol can be used further to generate rutin by glycosylation. Both catechin and epicatechin were the monomers of proanthocyanidin, which were synthesized by the catalyst of *LAR* and *ANR*, respectively. Low expression abundance genes are generally considered to be the limited step of a synthesis processes. This viewpoint is in accordance with our study. In the present study, most of annotated *LAR, ANR* and *FLS* unigenes were normally low or moderate-abundance, but their expressions were observed to be significantly up-regulated under cold stress. As evidence of the activity improvement of *LAR, ANR* and *FLS*, the contents of catechin, epicatechin and quercetin were remarkably increased to 5.02, 5.26, and 4.95 times, respectively.

## Conclusions

In summary, we surveyed the transcriptome of *T. hemsleyanum* and annotated a large number of DEGs involved in cold stress, which would provide a potential platform for future functional genomic research. Analysis of physiological index and DEGs revealed that the critical period of cold damages in *T. hemsleyanum* was from 12 h to 48 h. Most of the cold regulated DEGs were early-response gene. A total of 114 unigenes were assigned to the flavonoid biosynthetic pathway. 14 genes most likely to encode flavonoid biosynthetic enzymes were identified. Flavonols of *T. hemsleyanum* might play a crucial role in combating cold stress. Some key upstream genes (P*AL, 4CL*, and *CHS)* and some downstream genes for flavonols synthesis (*ANR, FLS*, and *LAR)* were induced by cold stress, which would result in a significant increase of crucial flavonol ingredients (catechin, epicatechin, rutin,and quercetin) in *T. hemsleyanum.*

The data presented here gives an insight into the molecular and physiological changes underlying the cold damage process in *T. hemsleyanum*, and the results can give a clue for identifying candidate genes as promising targets for genetic engineering and for the creation of novel germplasms with high cold-tolerance. Besides, the results provide valuable information for understanding the biosynthetic pathway of flavonoids in *T. hemsleyanum*. Additionally, the de novo transcriptome data would also enrich the plant database and eventually serve as reference sequences for other Vitaceae family members.

## Methods

### Plant materials and cold stress treatments

Healthy sterile test-tube plantlets of *T. hemsleyanum* were obtained and cultured as the same as our previous report [23]. In order to determine a rational sampling plan, the freezing experiments were conducted at − 8 °C, − 4 °C, 0 °C, and 4 °C for 0 h, 4 h, 8 h, 12 h, 24 h, 48 h, and 72 h, with three replications. Each sample was pooled from 10 seedlings. For each test temperature and time, physiological traits, including proline, malondialdehyde (MDA), soluble sugar and soluble protein content, and peroxidase (POD) activity were determined.

Based on the preliminary work results of physiological parameters determination, the temperature and sampling time of cold stress treatment were optimized. For both the control and stress tests, these seedlings were cultured in the same conditions except the different temperatures at 25 °C or 0 °C. After 0 h (control), 12 h (S1), 24 h (S2), and 48 h (S3) of cold stress treatment, the young leaves of 10 individual 4 week-old seedlings were collected and immersed in liquid nitrogen immediately and stored at − 80 °C for RNA extraction and quantitative real-time PCR analysis. Three biological replicates were performed at the same time. Each biological replicate consisted of a pool of leaves from 10 seedlings.

### Physiological parameters determination

POD activity was measured according to our previous report [24]. POD activity was determined based on the linear increase in absorbance at 460 nm during incubation of the extracts at 25 °C with 10 mM guaiacol and 1 mM H_2_O_2_ in 0.05 M sodiumacetate buffer (pH 5.4) in a total volume of 3 ml. An enzyme unit (U) was defined as the increase of absorbance by 0.01 per min. POD activity (U/g) = (∆A470 × V_t_) × (W × V_s_ × 0.01 × t)^− 1^; ∆A470:alteration of absorbance, W: fresh weight of leaves (g), V_t_: total volume of extractive enzyme (mL), V_s_: determined enzyme volume (mL),t: reaction duration (min),.

MDA content was measured as previously described [25]. MDA content (μmol/g) = (C_MDA_ × V_r_ × 10^− 3^ × V_t_) × (W × V_s_)^− 1^. C_MDA_ = 6.45(A_532_-A_600_)-0.56A_450_, μmol/L); V_r_: reaction volume (mL); V_t_: total volume of total extractive enzyme (mL), V_s_: tested enzyme volume (mL); W: the weight of leaves (g).

Soluble protein content was measured by Coomassie Brilliant Blue G-250 staining [25]. The absorbance of the supernatant was measured at 595 nm. Soluble protein content (mg/g) = C× V_t_ × (V_s_ × W × 1000)^− 1^; C: protein quantity determined from the standard curve (μg); V_t_: total volume of the extracted solution (mL); V_s_: sample solution volume (mL); W: the weight of samples (g).

Electrolyte leakage was determined by the method according to Fu et al. with some modifications [26]. The fresh leaves (1 g) were washed by deionized water and incubated with 15 ml deionized water at 25 °C for 24 h, and then initial electrolyte leakage (EC1) was measured. Then, Total electrolyte leakage (EC2) was calculated after that the samples were boiled for 20 min. The electrolyte leakage was expressed as percent (EL%) = EC1/ EC2 × 100%.

### RNA extraction and quality determination

Total RNA was extracted with plant RNA Reagent (Invitrogen) following the manufacturer’s protocol. RNA purity was checked using the kaiaoK5500®Spectrophotometer (Kaiao, Beijing, China). RNA integrity and concentration was assessed using the RNA Nano 6000 Assay Kit of the Bioanalyzer 2100 system (Agilent Technologies, CA, USA) at Annoroad Gene Technology (Beijing) co., LTD. mRNA was purified from total RNA using poly-T oligo-attached magnetic beads. RNA concentration of library was measured using Qubit® RNA Assay Kit in Qubit® 3.0. Insert size was assessed by the Agilent Bioanalyzer 2100 system (Agilent Technologies, CA, USA). The clustering was performed on a cBot cluster generation system using HiSeq PE Cluster Kit v4-cBot-HS (Illumina). After cluster generation, the libraries were sequenced on an Illumina HiSeq 2500 platform and 150 bp paired-end reads were generated. Low-quality RNA-Seq reads (Qscore < Q30) were discarded.

### De novo assembly and sequence clustering

The software Trinity was used for de novo assembly which was developed at the Broad Institute and the Hebrew University of Jerusalem. Trinity partitions the sequence data into many individual de Bruijn graphs, each representing the transcriptional complexity at a given gene or locus. Each graph was processed independently to extract full-length splicing isoforms and to tease apart transcripts derived from paralogous genes. The clean data were mapped to the assembled transcripts by Bowtie 2(v2.2.3). Homogeneity analysis, evaluation of the proportion of chimera, accuracy and core protein ratio were performed.

### Coding regions prediction and transcriptome functional annotation

The candidate coding regions within transcript sequences, such as those generated by de novo RNA-Seq transcript assembled by Trinity were identified by TransDecoder. The functional annotation of unigenes and ORFs were performed by Trinotate.

### RPKM estimation and DEG analysis

Reads Count for each gene in each sample was counted by HT Kilobase Millon Mapped Reads each sample, the formula is shown as:

$$ \mathrm{R}\mathrm{PKM}={10}^6\ast \mathrm{R}/\left(\mathrm{NL}/{10}^3\right) $$

R is the number of reads in a certain sample that is assigned to a certain gene, N is the total number of mapped reads in the certain sample and L is the length of the certain gene.

The differential gene expression between biological replicates was analyzed by DESeq(v1.16). *P*-value could be assigned to each gene and adjusted by the Benjamini and Hochberg’s approach for controlling the false discovery rate. “q≤0.05 and |log2_ratio|≥1” was set as the threshold to determine the differentially expressed genes (DEGs).

### Functional annotation and classification of DEGs

GO enrichment analysis could reveal the biological functions of the DEGs. The GO (Gene Ontology, http://geneontology.org/) enrichment of DEGs was implemented by hypergeometric test. GO terms with q < 0.05 were considered to be significantly enriched. KEGG (Kyoto Encyclopedia of Genes and Genomes http://www.kegg.jp/) analysis of the DEGs was performed to identify the associated biochemical and signal transduction pathways. KEGG terms with q < 0.05 were considered to be significantly enriched.

### Quantitative real time PCR analysis

Quantitative real-time RT-PCR (qPCR) was performed to examine expression patterns of the candidate unigenes using a SteponePlus real-time PCR system (Applied Biosystems, Forster City, CA, USA). The primers for amplification are listed in Table 2. There primers were designed using Primer3 (http://simgene.com/Primer3) and synthesized by Invitrogen. Each reaction contained 2 μl of cDNA, 10 μl of 2x Power SYBR green PCR master mix (Applied Biosystems, Forster City, CA, USA), and 2 μl of forward and reverse primers in a final volume of 20 μl. The PCR reactions were conducted by incubation at 95 °C for 3 min followed by 40 cycles of 95 °C for 15 s and 60 °C for 45 s. A relative mRNA level was calculated by the 2^-ΔΔCt^, using the actin gene as an endogenous control. Dissociation-curve analysis was carried out to confirm the amplification specificity. Three technical replicates were performed for each sample and the data are shown as means ± standard errors (SE) (*n* = 3).

**Table 2:** List of primer sequences of randomly selected unigenes used for qRT-PCR analysis

Gene ID	Tm	Primer F (5′-3′)	Primer R (5′-3′)
c99496_g1	56.5	GAAAAACCCAGACTCAATCCCA	ACCAAGCTTCTCAATCACAACG
c71418_g1	58.5	ACCTCGGAGAAGGGTGGAC	AACAAGGATTGGGGGGAAA
c96971_g1	53.0	TACTACTTCCGCATCACCAAC	GGACTTCAACGACCACCATAT
c81457_g1	56.0	TTTGCTGAGATTATTATTTGTGGG	AAGATTAGGGTAAGGTTAGCGTGT
c100796_g1	60.5	GGTTTGTTGAAGCAGAAGGCG	GGTGTTAGGGAAGGTGAGGGC
c101069_g2	57.3	AGAGAGAAAGCGATAATGGAAGG	GTTGAAGGCGAGCGTAGGTG
c85087_g1	52.5	CAATTACAATTCACATTCAACAA	ATAGCATCCCTACCATTACAAAC
c77820_g1	53.0	AGAAAGAAATCCCTAAAGGGG	AGGAATGCTGTGTAGCACAAC
c101781_g1	53.0	AGAGAATAATGCAGGAGCACG	GCTGAGACAAGTTGGAAGAGTG
c89063_g2	57.8	TGCTACTTCCCCCACTGCTG	ACACTCCCCTTGTTCCACCC
c87939_g1	55.0	GGGAAAAAGACTGAAGAGAAAATAG	GAGAAAGGTAAGCTGAAACAAAATC
c98652_g1	57.5	GCAGTAGTGGGACGAGACAGG	GAGGGGAGTAGATGGGTGGAG

### Qualitative and quantitative analysis of flavonols

About 0.5 g freeze-dried sample was extracted in 20 mL 90% (v/v) methanol solution using sonication at 4 °C for 60 min. The mixture was centrifuged at 10000 *g* for 15 min and passed through a 0.22 μm filter, after which the volume was adjusted to 1 mL with methanol solution. All samples were separated by Agilent SB-C18 (4.6 × 250 mm, 5 μm) column at 30 °C in a Diane U-3000 HPLC system equipped with a DAD detector (Diane Technologies, USA). The mobile phase was acetonitrile (solvent A) and 0.1%(v/v) phosphoric acid in H_2_O (solvent B). The gradient elution program was as follows: 0–30 min, 10–25% A; 30–40 min, 25–95% A; 40–45 min,95% A; 45–60 min, 95–100% A; The flow rate was 0.8 mL.min^− 1^ and the injection volume was 10 μL. The monitoring wavelength was set at 230 nm. The peak areas of the seven flavonol compounds; quercetin procyanidine B1 procyanidine B2 catechin epicatechin rutin kaempferol were approximately calibrated by using quercetin (Sigma-Aldrich, St Louis, MO, USA) standard. The seven flavonol compounds were identified and quantified based on their spectra, standards. The content of each flavonol was presented as milligrams of quercetin equivalent (QE) mg·g^− 1^ of DW. All experiments were repeated three times.

### Statistical analysis

All experiments were carried out in triplicate, and the results were expressed as means± standard deviation (SD). The data were subjected to one-way analysis of variance in SPSS 17.0 for Windows (SPSS Inc., USA), and the significance between treatments was assessed by LSD multiple comparison test at *P* < 0.05.

## Additional files

Additional file 1: Length Hist Distribution of Trinity and Unigene in *T. hemsleyanum* transcriptome. There were 151,924 ‘Trinity’ genes and 106,275 unigenes, which ranged from 201 to 15,668 bp in length. The average length of unigenes was 676 bp. The N50 and N90 length was 1121 bp and 262 bp, respectively. (XLS 484 bytes)

Additional file 2: Unigenes Expression matrix data in *T. hemsleyanum* transcriptome. Differential gene expression levels under cold stress and functional classifications of all annotated unigenes in *T. hemsleyanum* transcriptome. (XLSX 25195 kb)

## Data Availability

The datasets generated during the current study are available in Additional file 1 Length Hist Distribution of Trinity and Unigene in *T. hemsleyanum* transcriptome Additional file 2 Expression matrix data of genes of *T. hemsleyanum* during the cold treatment.

## Abbreviations

4CL: 4-coumarate CoA ligase
ANS: anthocyanidin synthase
C4H: cinnamic acid 4-hydroxylase
CHI: chalcone isomerase
CHS: chalcone synthase
DFR: dihydroflavonol reductase
F3′H: flavonoid 3′-hydroxylase
F3H: flavanone-3-hydroxylase
MDA: ZMalondialdehyde
PAL: phenylalanine ammonia lyase
POD: Peroxidase
UFGT: UDP-glucose: flavonoid-3-O-glucosyltransferase
